# Identifying Environmental Endocrine Disruptors Associated With the Age at Menarche by Integrating a Transcriptome-Wide Association Study With Chemical-Gene-Interaction Analysis

**DOI:** 10.3389/fendo.2022.836527

**Published:** 2022-02-24

**Authors:** Mengnan Lu, Ruoyang Feng, Yujie Qin, Hongyang Deng, Biyao Lian, Chunyan Yin, Yanfeng Xiao

**Affiliations:** ^1^Department of Pediatrics, The Second Affiliated Hospital of Xi’an Jiao Tong University, Xi’an, China; ^2^Department of Joint Surgery, HongHui Hospital, Xi’an Jiao Tong University, Xi’an, China

**Keywords:** menarche, puberty, environmental endocrine disruptor, GWAS, TWAS, CGSEA

## Abstract

Menarche is the first occurrence of menstrual bleeding and one of the most important events of female puberty. Alarmingly, over the last several decades, the mean age at menarche (AAM) has decreased. Environmental endocrine disruptors (EEDs) are chemicals that may interfere with the endocrine system, resulting in adverse developmental, immunological, neurological, and reproductive effects in humans. Thus, the effects of EEDs on fertility and reproduction are growing concerns in modern societies. In this study, we aimed to determine the influence of genetic and environmental factors on AAM. We used data from an AAM genome-wide association study of 329,345 women to conduct a transcriptome-wide association study (TWAS) with FUSION software. As references, we determined the gene-expression levels in the hypothalamus, pituitary gland, ovaries, uterus, and whole blood. We performed Gene Ontology and Kyoto Encyclopedia of Genes and Genomes enrichment analyses using the significantly dysregulated genes identified by the TWAS. Using the STRING database, we also generated a protein–protein-interaction network to analyze common AAM-specific genes identified by the TWAS with different tissues. We performed chemical-related gene set enrichment analysis (CGSEA) and identified significant TWAS genes to uncover relationships between different chemicals and AAM. The TWAS identified 9,848 genes; among these, 1580 genes were significant (*P* < 0.05), and 11 genes were significant among the hypothalamus, pituitary, ovary, uterus, and whole blood. CGSEA identified 1,634 chemicals, including 120 chemicals significantly correlated with AAM. In summary, we performed a TWAS (for genetic factors) and CGSEA (for environmental factors) focusing on AAM and identified several AAM-associated genes and EEDs. The results of this study expand our understanding of genetic and environmental factors related to the onset of female puberty.

## Introduction

Puberty is a complex process occurring between childhood and adulthood, producing internal and external physical changes that promote the development of primary and secondary sexual characteristics important for sexual reproduction (1). Sex hormones are responsible for the physical manifestations of female puberty, including thelarche, pubarche, and menarche (2). Menarche is the first menstrual bleeding and one of the most important events of female puberty. Over the last several decades, the mean age at menarche (AAM) has declined, which is concerning (3). Epidemiological evidence suggests that the onset of puberty is advancing in humans through undetermined mechanisms (4).

Environmental endocrine disruptors (EEDs) are chemicals that interfere with the endocrine system and evoke adverse developmental, immunological, neurological, and reproductive effects in humans (5). EEDs are common in human living environments and include pesticides, plasticizers, industrial by-products, drugs, and some naturally occurring phytochemicals (6). These exogenous chemicals can interfere with the complex endocrine system, causing adverse health effects, such as reproductive disorders, metabolic diseases, and various cancers (7). Chronic exposure to EEDs may play a role in accelerating or delaying the onset of menarche, and extensive research has shown that pesticides, phenols, polycyclic aromatic hydrocarbons, phthalates, and some heavy metals are responsible for hormone metabolism disorders occurring during puberty (8). Consequently, the effects of EEDs on fertility and reproduction are a growing concern in modern societies (9).

Recent genome-wide association studies (GWASs) have identified thousands of genetic variants associated with complex phenotypes and have provided insights into their genetic architectures. GWASs are also extremely well-suited for identifying common single-nucleotide polymorphism (SNP)-based variants (10). GWAS analysis has been conducted to research early puberty and identify the genetic characteristics of idiopathic central precocious puberty and validate the polygenic risk for early puberty (11). Felix R Day et al. have identified 389 independent, genome-wide significant signals for AAM, which explained ~7.4% of the population variance in AAM and corresponded to ~25% of the estimated heritability (11).

Genetic loci cause trait variations, ranging from growth and fitness in simple organisms to disease in humans. Determining the genetics of gene-expression differences has emerged as a key approach for linking DNA-sequence variations to phenotypes (12). Transcriptome-wide association study (TWAS) analysis has been used to identify significant expression-trait associations by integrating genotypes, gene-expression levels, and phenotypes in order to gain insights into the genetic basis of complex traits (13). In a recent study, a TWAS was performed to discover transcriptome differences that affect the age of natural menopause (ANM), and 34 ANM-associated genes were reported (14). The hypothalamic–pituitary–gonadal axis controls puberty and reproduction (15). Therefore, investigating the associated gene-regulation relationships may help identify important genes that are co-expressed in all tissue types during AAM.

Here, we aimed to determine the influences of genetic and environmental factors on AAM by performing a large-scale TWAS for AAM based on a GWAS data set. We investigated gene-expression levels in the hypothalamus, pituitary gland, ovaries, uterus, and whole blood. We also reevaluated the expression of TWAS-identified genes, functionally explored the genes, and identified AAM-associated EEDs.

## Methods

### Summary of the AAM GWAS Data Used in This Study

We used published GWAS summary data for female AAM (16). Briefly, Day et al. performed a meta-analysis of pooled GWAS data from multiple studies of 329,345 women of European ancestry, including 40 studies from the ReproGen consortium (*N* = 179,117), 23andMe (*N* = 76,831), and the United Kingdom Biobank (*N* = 73,397). The results were grouped into the relatively sparse HapMap 2 reference panel or attributed to the 1000 Genomes Project reference panel using gene-centric arrays. In each study, the associations of SNPs with AAM were based on a two-tailed additive linear-regression model and several factors, including the age at the study visit and other study-specific covariates. Day et al. performed an expanded genomic analysis of AAM in women that was nearly three times larger and used denser genomic data than previous studies. Information on the subjects, genotypes, responsibilities, and quality control were detailed in the published study (16). In addition, ethical approval was not applicable for this study as publicly available data were used for the analysis.

### TWAS Analysis

We used FUSION (13) software (http://gusevlab.org/projects/fusion/) to analyze the GWAS summary data for the previous meta-analysis of AAM. The most popular TWAS methods, such as PrediXcan, TWAS-Fusion, and SMR, test causal relationships between gene-expression levels and complex traits (17), among which, the TWAS-Fusion method is used more often. Briefly, Bayesian sparse linear-mixed models (18) were used to calculate SNP expression weights for specific genes at the 1-Mb cis position and estimate the association of predicted expression levels with AAM using the following formula: Ztwas = w + Z/(w × [Lw]^1/2^) (13), where w denotes the weight, Z denotes the Z-score, and L denotes the SNP correlation matrix (definition, LD). We used the gene-expression weights for the hypothalamus (N samples = 108; N features = 2,315), pituitary (N samples = 157; N features = 4,402), ovary (N samples = 122; N features = 2,809), uterus (N samples = 101; N features = 2,135), and whole blood (N samples = 1,264; N features = 4,701) as references, which are available for download from the FUSION website (http://gusevlab.org/projects/fusion/). We estimated the transcriptome-wide significance as P = 5.08 × 10^−6^ (0.05/9,848) using the Bonferroni correction (14). Manhattan plot was made by “CMplot”(v. 3.6.2) in R package.

### Functional Exploration of Genes

We performed Kyoto Encyclopedia of Genes and Genomes (KEGG) (19) and Gene Ontology (GO) (20) enrichment analyses to identify and confirm related biological processes. KEGG and GO enrichment were performed using the R packages “org.Hs.eg.db” and “clusterProfiler” (R Foundation for Statistical Computing, Vienna, Austria. https://www.R-project.org/).

### Interaction-Network Analysis

We generated a protein–protein-interaction (PPI) network using the STRING database, v11.5 (STRING, https://string-db.org), requiring a confidence score of 0.15 and “active interaction sources” based on a previous study (21). Cytoscape (22) was used to visualize all interaction networks, and the Molecular Complex Detection (MCODE) plugin (23) was used for module analysis.

### Chemical Gene Expression Annotation Data Set

The chemical-related gene-expression annotation data set used in this study was downloaded from the Comparative Toxicology Genomics Database (CTD) (http://ctdbase.org/downloads/). The CTD mainly provides four data sets, including a chemical–gene-interaction function, a chemical–disease association, a genetic disease association, and a chemical element-phenotypic association. The CTD integrates the four data sets to automatically construct a hypothetical chemical–gene phenotype disease network to illustrate the molecular mechanisms underlying diseases that affect the environment (24). Cheng et al. downloaded and used 1,788,149 chemical–gene pair annotation terms for humans and mice, generating 11,190 chemical substance-related gene sets (25). We also used that data set to perform our chemical-related gene set enrichment analysis (CGSEA).

### CGSEA

CGSEA is a flexible tool for assessing associations between chemicals and complex diseases. Briefly, the software uses genome-wide summary data (e.g., a summary of the TWAS data and messenger RNA [mRNA]-expression profiles) to explore functional relationships among chemical substances and diseases from a genomics perspective, for many complex diseases and characteristics. We used the CTD Chemical Gene Interaction Network and TWAS Expression Association to test the AAM statistics and weighted Kolmogorov–Smirnov running sum statistics to explore the relationships between chemicals and AAM, as described previously in greater detail (26). Specifically, in this study, we performed 10,000 permutations to obtain the empirical distribution of the gene set enrichment analysis (GSEA) statistical data for each chemical and then calculated the *P*-value of each chemical based on the empirical distribution of the CGSEA statistical data. Based on previous findings, we excluded gene sets containing <10 or >200 genes to limit the influence of gene set sizes on the results (27). To avoid deviations from expression correlations between genes, a package of lme4qtl software (28) was used to adapt the mixed-model regression of the TWAS Z-score based on the number of members in each gene set, in order to consider the correlation of the Z-score between genes caused by LD (28). A detailed description of the analytical method used was provided previously (25).

## Results

### TWAS Analysis of AAM

**Figure 1:**
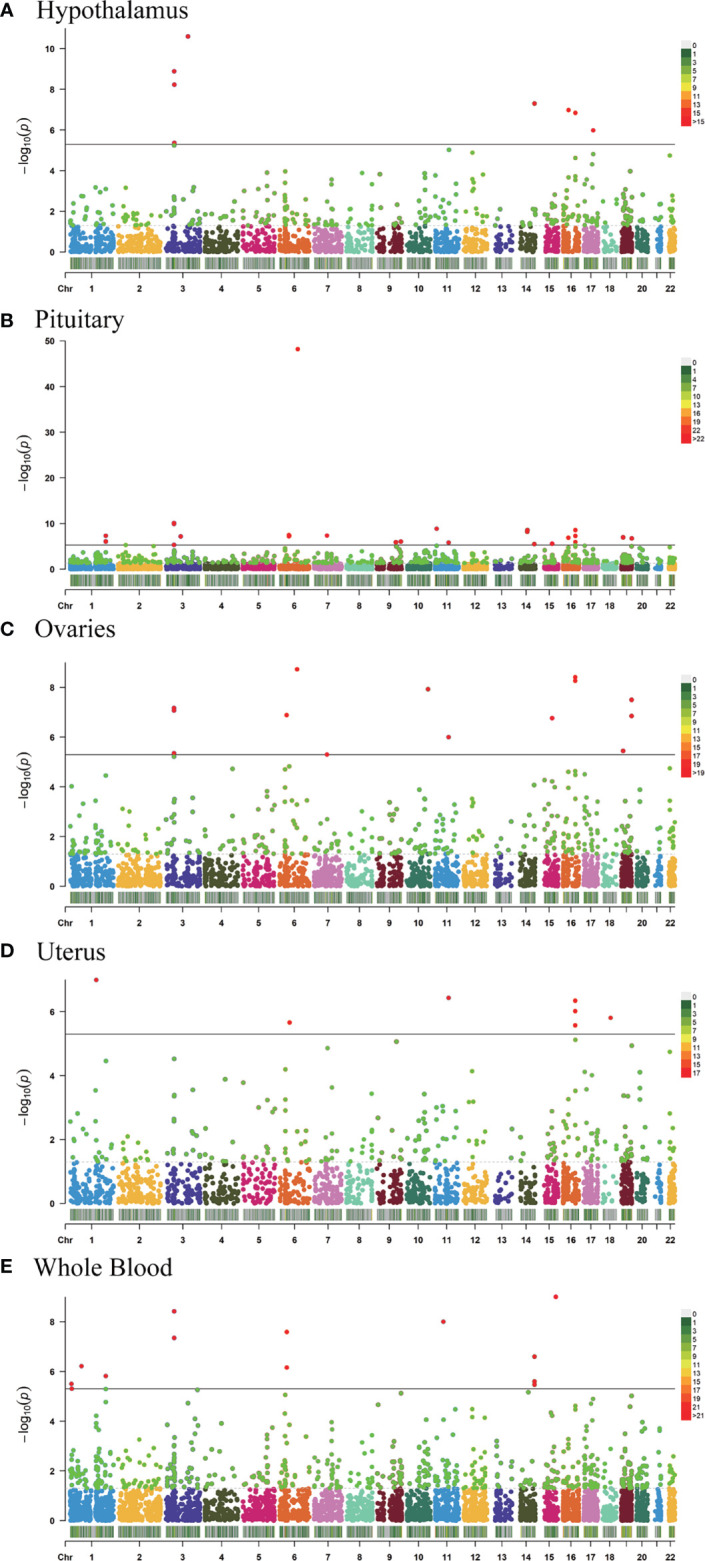
Manhattan plots of the association results from the AAM TWAS. The dashed horizontal lines represent P = 5.00 × 10^−2^. The solid horizontal lines represent P = 5.00 × 10^−6^ (Bonferroni correction). Each dot represents the genetically predicted expression of one specific gene in the hypothalamus, pituitary gland, ovary, uterus, and whole blood tissues. The X axis represents the chromosome (Chr) encoding the corresponding gene, and the Y axis represents the negative logarithm of the association *P _TWAS_* value. **(A)** Gene-expression weights for the hypothalamus. **(B)** Gene-expression weights for the pituitary gland. **(C)** Gene-expression weights for the ovaries. **(D)** Gene-expression weights for the uterus. **(E)** Gene-expression weights for whole blood.

TWAS analysis identified 9,848 genes from the GWAS summary data and of those 1,580 genes expression was associated with AAM (P <0.05) while 64 showed a significant association (P <5.08 × 10^−6^); 2,289 genes tested in hypothalamus and of 322 genes expression was associated with AAM (P <0.05) while 8 showed a significant association (P <5.08 × 10^-6^; **Figure 1A**), 4,362 genes tested in pituitary and of 568 genes expression was associated with AAM (P <0.05) while 26 showed a significant association (P <5.08 × 10^−6^; **Figure 1B**), 2,768 genes tested in ovary and of 366 genes expression was associated with AAM (P <0.05) while 14 showed a significant association (P <5.08 × 10^−6^; **Figure 1C**), 2,104 genes tested in uterus and of 253 genes expression was associated with AAM (P <0.05) while 7 showed a significant association (P <5.08 × 10^−6^; **Figure 1D**), and 4,671 genes tested in whole blood and of 604 genes expression was associated with AAM (P <0.05) while 9 showed a significant association (P <5.08 × 10^−6^; **Figure 1E**), respectively (**Supplementary Information**).

### Functional Exploration of TWAS-Identified Genes Associated With AAM

**Figure 2:**
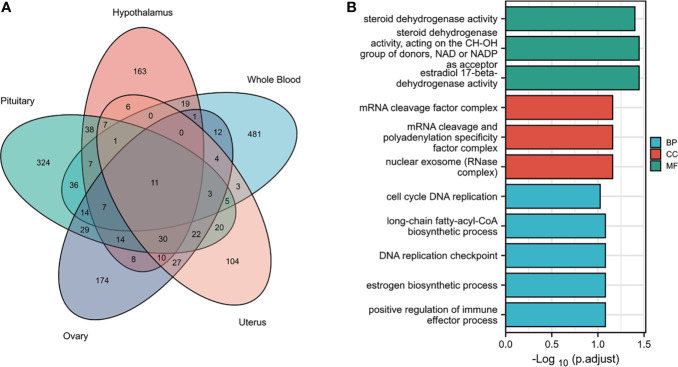
Functional exploration of the TWAS-identified genes associated with AAM. **(A)** Venn diagram revealing the overlap of TWAS-significant genes in different tissues. Red, hypothalamus; green, pituitary gland; purple, ovaries orange, uterus; blue, whole blood. **(B)** Bar plot of enriched GO terms for the overlapping genes.

Tissues have unique gene-expression profiles. Thus, we performed an overlap analysis of the significant genes in different tissues to identify the most representative genes. **Figure 2A** shows the resulting Venn diagram, which indicates the number of genes expressed in one or more tissues. Overall, 163 TWAS-identified significant AAM-specific genes were associated with the hypothalamus: 38 significant genes were associated with the hypothalamus and pituitary gland; 7 significant genes were associated with the hypothalamus, pituitary, and ovary; and 11 significant genes were associated with the hypothalamus, pituitary, ovary, uterus, and whole blood. The 11 novel TWAS-significant AAM-susceptible genes identified in all five tissues were *RBM6* (RNA-binding motif protein 6; chromosome 3), *PILRB* (paired immunoglobin-like type 2 receptor beta; chromosome 7), *CPSF1* (cleavage and polyadenylation-specific factor 1; chromosome 8), *INPP5E* (inositol polyphosphate-5-phosphatase E; chromosome 9), *MRPL43* (mitochondrial ribosomal protein L43; chromosome 10), *HSD17B12* (hydroxysteroid-(17-β)-dehydrogenase 12; chromosome 11), *TIPIN* (TIMELESS-interacting protein; chromosome 15), *FLYWCH1* (FLYWCH-type zinc finger 1; chromosome 16), *EXOSC6* (exosome component 6; chromosome 16), *ADORA2B* (adenosine A2b receptor; chromosome 17), and *SPATA20* (spermatogenesis-associated 20; chromosome 17). **Table 1** presents detailed information regarding these 11 genes, including the rsIDs of the most significant (i.e., best) GWAS SNPs in the locus (i.e., BEST.GWAS.ID) and the TWAS *P*-value (i.e., *P _TWAS_*).

**Table 1:** Significant TWAS-identified genes associated with AAM in all five tissues studied.

Gene	BEST.GWAS.ID	*P _TWAS_*				
		Hypothalamus	Pituitary gland	Ovary	Uterus	Whole blood
RBM6	rs3905330	0.00271	0.00009	0.00041	0.00041	0.00303
PILRB	rs2950520	0.02887	0.03400	0.02900	0.03800	0.03621
CPSF1	rs35253356	0.00528	0.01188	0.01056	0.02224	0.01915
INPP5E	rs10448340	0.03503	0.02700	0.01299	0.04100	0.01770
MRPL43	rs11190901	0.00014	0.00017	0.00030	0.00038	0.00070
HSD17B12	rs6485443	0.00272	0.00061	0.00316	0.00089	0.00368
TIPIN	rs2113688	0.00420	0.00902	0.00147	0.00420	0.02590
FLYWCH1	rs1834026	0.01500	0.01500	0.00772	0.01210	0.02630
EXOSC6	rs7196842	0.00019	0.00075	0.00003	0.00001	0.00002
ADORA2B	rs178837	0.01740	0.00027	0.00027	0.00027	0.00110
SPATA20	rs989128	0.02210	0.04920	0.03560	0.00639	0.00593

We subjected the TWAS-identified genes to GO analysis (**Figure 2B**). Five enriched GO terms belonged to the biological process (BP) category, including cell cycle DNA replication, long-chain fatty-acyl-CoA metabolic process, DNA replication checkpoints, estrogen biosynthetic processes, and positive regulation of immune effector processes. Three significantly enriched GO terms belonged to the cellular component (CC) category, including the mRNA cleavage factor complex, mRNA cleavage and polyadenylation specificity factor complex, and nuclear exosome (RNase complex). In terms of the molecular function (MF) category, the enriched GO terms primarily involved estrogen metabolism (such as steroid dehydrogenase activity or steroid dehydrogenase activity acting on the CH-OH group of donors), nicotinamide adenine dinucleotide or nicotinamide adenine dinucleotide phosphate as an acceptor, and estradiol 17-beta-dehydrogenase activity.

### PPI Network of the TWAS-Identified Genes

**Figure 3:**
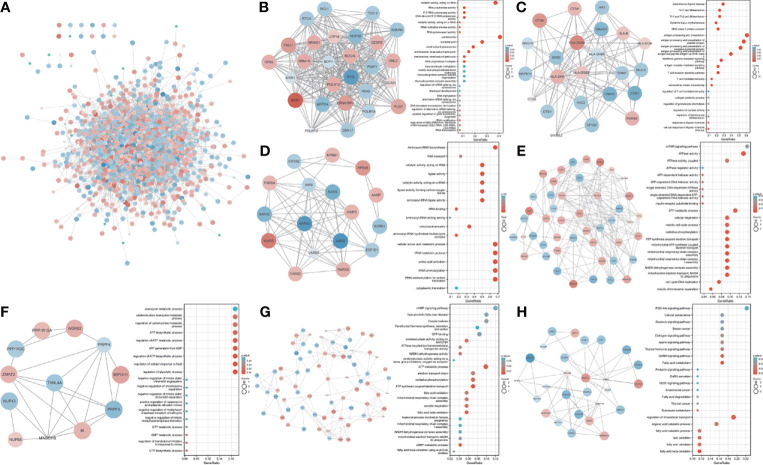
PPI network and significant modules. Red and blue circles indicate upregulated and downregulated TWAS-identified genes. **(A)** PPI network of the TWAS-identified genes. **(B-H)** Significant modules 1 to 7 of the PPI network and their functional exploration. GeneRatio: the ratio of the number of genes associated with a term linked to an enriched gene to the total number of enriched genes.

We used 1,580 TWAS-significant AAM-associated genes for PPI analysis and successfully transformed 1,056 protein-coding genes (**Figure 3A**). To effectively find densely connected regions of the PPI network, we formed seven MCODE clusters with the PPI network genes (**Figures 3B–H**). The hub genes identified using the MCODE plugin were further analyzed for functional exploration. MCODE cluster 1 (MCODE1) was related to ribosome biogenesis, female pregnancy, and blastocyst development. MCODE2 was characterized by genes in the human leukocyte antigen (*HLA*) family associated with the immune process. MCODE3, MCODE4, and MCODE5 were related to tRNA, energy metabolism processes, and biosynthetic processes. MCODE6 was associated with lipid oxidation and maternal processes involved in female pregnancy. MCODE7 contained some important significantly enriched terms, including fatty acid metabolic, gonadotropin-releasing hormone (GnRH), estrogen, oxytocin, and PI3K-Akt signaling pathways and breast cancer.

### CGSEA of the TWAS-Identified Genes

**Figure 4:**
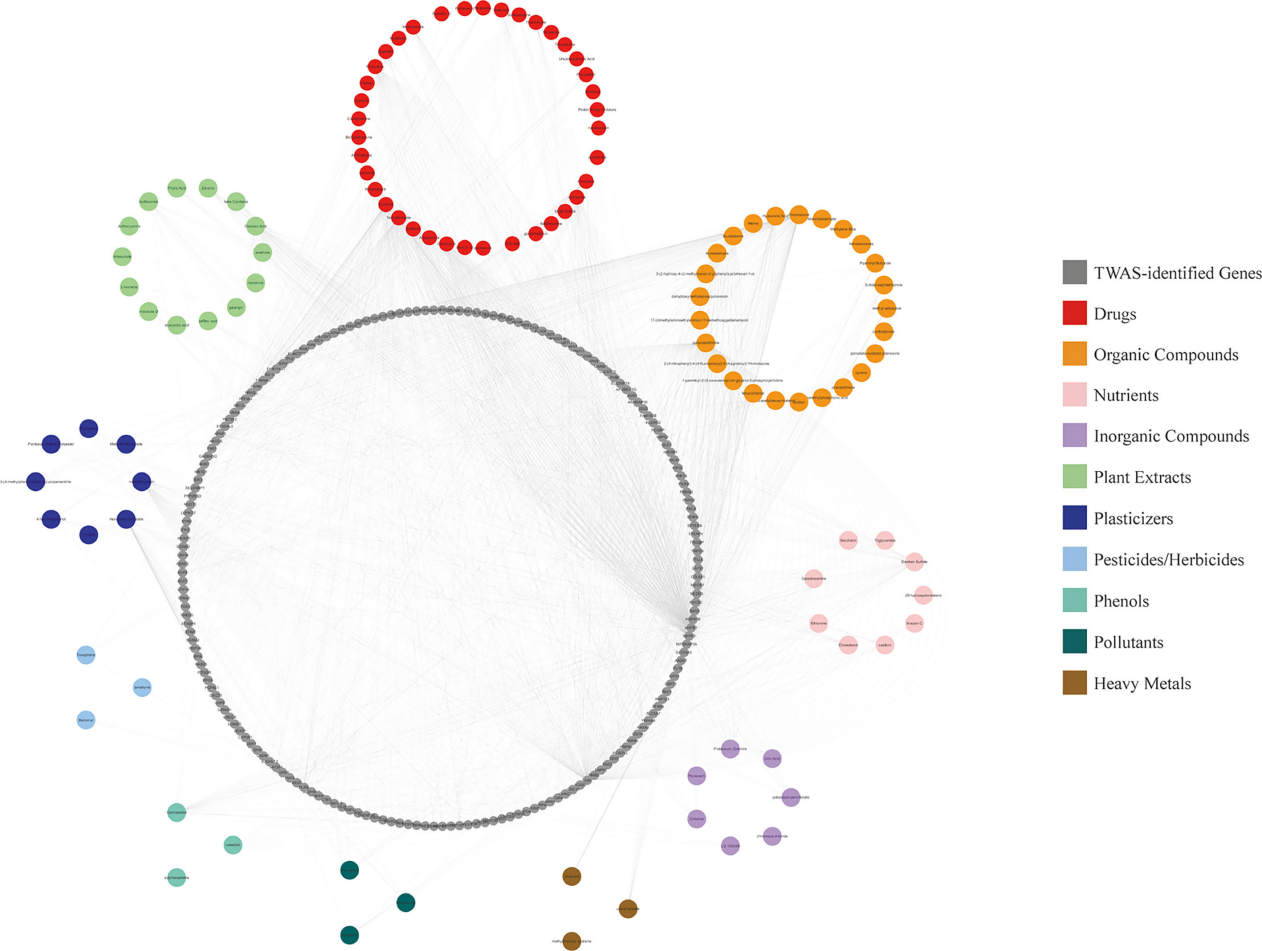
CGSEA results. Network of EEDs and their target genes, based on the TWAS-identified genes.

We performed CGSEA to investigate environmental factors that influence the onset of puberty, which identified 1,634 chemicals, including 120 chemicals that correlated significantly with AAM. These significant chemicals included some drugs (e.g., fluoxetine), pesticides (e.g., ametryne), plant extracts (e.g., isoflavones), nutrients (e.g., cholesterol), phenols (e.g., cannabidiol), phthalates (e.g., monobutyl phthalate), heavy metals (e.g., uranium), and atmospheric pollutants (e.g., phosgene). **Figure 4** illustrates our constructed network of EEDs and their target genes based on the TWAS-identified genes. Chemical with absolute normalize enrichment score (NES) values of >1 were considered significantly enriched according to GSEA. We identified 77 significantly enriched chemicals with an |NES| value of >1 and a P-value of <0.05 (**Table 2**).

**Table 2:** Significant chemicals identified by CGSEA of the significant TWAS-identified AAM-associated genes.

Chemical name	NES	P	Chemical name	NES	P
Chlorine	12.35459	0.00050	Acetaldehyde	3.24524	0.01399
Fluoxetine	8.68168	0.00050	Heliotrine	2.50818	0.01449
Proton pump inhibitors	63.99450	0.00050	Pseudocumene	3.38592	0.01499
Fexofenadine	18.12249	0.00100	Chlordecone	2.98902	0.01499
Torcetrapib	7.28264	0.00150	Gefitinib	1.74158	0.01549
Methylmethacrylate	6.88844	0.00250	Cholesterol	2.32618	0.01599
Chromous chloride	8.16988	0.00350	N-nitroso-tris-chloroethylurea	2.92314	0.01649
Thalidomide	4.83989	0.00350	Methylmercury cysteine	1.12554	0.01799
Pentosan sulfuric polyester	5.33529	0.00400	Pyrene	2.66545	0.01799
Anacardic acid	4.53182	0.00450	Dehydroxymethylepoxyquinomicin	1.58757	0.01799
Calcimycin	5.92628	0.00450	Casticin	2.55138	0.01899
Palbociclib	2.05821	0.00550	Squalestatin 1	2.60788	0.01949
Temozolomide	4.66128	0.00550	Periodate-oxidized adenosine	2.80821	0.01999
Uranyl acetate	3.30552	0.00600	Isoflavones	2.19951	0.02049
Catechol	4.40109	0.00650	Melphalan	2.67401	0.02099
Cannabidiol	3.31796	0.00650	Clomipramine	1.57621	0.02149
Amitriptyline	4.15778	0.00700	Uranium	2.49394	0.02199
Ibuprofen	3.81067	0.00700	Monobutyl phthalate	2.52550	0.02249
Uric acid	2.27066	0.00700	Rimonabant	2.71940	0.02349
Methylparaben	5.83928	0.00750	Toxaphene	1.21501	0.02399
Oleoyl-estrone	1.50555	0.00750	Ursodeoxycholic Acid	2.44936	0.02399
2-(4-nitrophenyl)-4-(4-fluorophenyl)-5-(4-pyridinyl)-1H-imidazole	1.72254	0.00750	Letrozole	1.83199	0.02449
Benzbromarone	3.93596	0.00750	4-toluidine	2.47743	0.02499
Ethionine	4.06789	0.00750	Carcinogens	2.27375	0.02649
SK&F 83959	3.56873	0.00800	Okadaic acid	2.35105	0.02649
Potassium perchlorate	3.55962	0.00850	Sulfasalazine	1.98058	0.02749
Phenanthrene	3.09849	0.00900	Pyrazolanthrone	2.25782	0.02899
SNX 2112	1.69629	0.00900	Pravastatin	1.71224	0.03098
Aripiprazole	1.82475	0.00950	Piperonyl butoxide	2.13714	0.03298
Asbestos	3.88868	0.00950	Potassium chloride	1.23405	0.03298
S-Adenosylmethionine	2.34165	0.00950	Bucladesine	1.80893	0.03498
Reserpine	2.09526	0.01049	3-(2-hydroxy-4-(2-methylnonan-2-yl) phenyl) cyclohexan-1-ol	2.16625	0.03598
Ciprofibrate	3.59698	0.01099	Caffeic acid	1.43915	0.04248
Probenecid	2.54975	0.01099	Methimazole	1.97181	0.04248
Pyrogallol	3.56596	0.01099	Methyl cellosolve	1.86438	0.04298
Homocysteine	3.54662	0.01149	Estrogens	1.47629	0.04448
Phosgene	3.01541	0.01199	Ciglitazone	1.26701	0.04598
Tebuconazole	2.51505	0.01349	Hyaluronic acid	1.84193	0.04698
Paraoxon	2.58233	0.01349			

## Discussion

Puberty is a developmental period affecting the body and behavior of children developing secondary sexual characteristics (29). Pubertal hormones play important roles in the adrenal, gonadal, and growth axes (30). Pubertal timing is determined by the activation of pulsatile hypothalamic GnRH secretion when pituitary gonadotropin secretion and downstream gonadal maturation are also initiated (31). Thus, various tissue types are affected by AAM, including the hypothalamus, pituitary gland, ovaries, uterus, and whole blood.

Up to now, previous studies have identified several genes associated with AAM, these results are similar to our studies (**Table 3**). Here, we performed a comprehensive TWAS to evaluate the relationship between AAM and predicted genes found in the hypothalamus, pituitary gland, ovaries, uterus, and whole blood. We identified 11 genes whose genetically predicted expression was associated with AAM (*P _TWAS_* ≤ 0.05), including 10 novel genes (*RBM6*, *PILRB*, *CPSF1*, *INPP5E*, *MRPL43*, *TIPIN*, *FLYWCH1*, *EXOSC6*, *ADORA2B*, and *SPATA20*) and one gene (*HSD17B12*) linked to AAM in a previous GWAS. The 11 protein-coding genes identified in our study have been implicated in estrogen metabolism. We also identified enriched fatty acid-metabolism pathways, similar to the findings of a recent study (36), indicating that different types of fatty acids may influence puberty timing. Additionally, individual fatty acids might have different physiological and metabolic effects, including the progression of pubertal development. Therefore, our study provides new information that improves our understanding of the genetics and etiology of AAM.

**Table 3:** Previously reported significant AAM-related genes.

Authors	SNP	Gene	P^a^	*P_TWAS_^b^*
John RB Perry, et al. (32)	rs10148448	MEG3	2.10E-04	2.52E-07
Diana L. Cousminer, et al. (33)	rs12917823	MAPK3	P < 5.00E-02	1.22E-03
	rs17046434	ADCY3	P < 5.00E-02	2.42E-02
	rs7759938	LIN28B	P < 5.00E-02	6.37E-49
Cathy E. Elks, et al. (34).	rs7759938	LIN28B	1.60E-58	6.37E-49
	rs10148448	BEGAIN	1.70E-10	2.55E-06
	rs4929923	TRIM66	2.40E-08	1.38E-09
	rs3905330	RBM6	1.40E-09	8.60E-05
	rs633715	SEC16B	1.50E-09	4.07E-03
	rs4955420	KLHDC8B	1.80E-09	6.13E-06
	rs16938437	PHF21A	1.40E-09	9.98E-09
	rs2687729	EEFSEC	1.00E-08	2.51E-11
	rs8104651	OLFM2	4.60E-10	1.10E-07
	rs939317	ECE2	2.30E-09	4.89E-03
	rs12641981	GNPDA2	8.70E-08	3.85E-02
	rs12056794	MSRA	2.40E-02	1.00E-02
Nicholas Mancuso, et al. (35).	rs6580698	CCDC65	P < 5.00E-08	7.30E-05
	rs7330016	COG6	P < 5.00E-08	4.40E-02
	rs12917823	INO80E	P < 5.00E-08	2.47E-05
	rs3761919	NUCKS1	P < 5.00E-08	4.86E-08
	rs4717903	PMS2P5	P < 5.00E-08	6.58E-04
	rs3761919	RAB7L1	P < 5.00E-08	9.47E-07
	rs4717903	STAG3L2	P < 5.00E-08	4.37E-08
	rs2274351	TMEM180	P < 5.00E-08	2.24E-04

a P values of previously reported AAM-related genes.

b Significant TWAS-identified genes associated with AAM in present research.

*HSD17B12* encodes 17β-hydroxysteroid dehydrogenase (17β-HSD), which is crucial for converting estrone into estradiol and fatty acid elongation. Our results support those of Kemiläinen et al. (37), who found that *HSD17B12* played a vital role in female fertility through arachidonic acid metabolism. Specifically, an investigation of the 17β-HSD enzyme expression in human and mouse ovaries revealed that female *HSD17B*^+/-^ mice more often had frequent dysfunctional oogenesis and ovulation, leading to less frequent births. Under extreme circumstances, haploinsufficiency of the *HSD17B12* gene in female mice resulted in subfertility. *HSD17B12* can affect fatty acid elongation and ceramide accumulation in the serum (38). Previous data showed that central ceramides participate in the timing of female puberty (39), suggesting that *HSD17B12* could be a candidate gene for female puberty. Notably, *HSD17B12* was also related to endometrial (40), ovarian (41), and breast (42) cancers.

Previous results have shown that INPP5E is concentrated at the cilia base, where it helps control phosphoinositide metabolism (43). Primary cilia are present on virtually all cell types. Cilia appear to exert a crucial modulatory role in appropriate axonal wiring due to INPP5E-dependent activation of the PI3K–AKT signaling pathway, triggering an axonal Ca^2+^ wave (44). GnRH neurons in adults are multi-ciliated, and the percentage of GnRH neurons possessing multiple Kiss1r-positive cilia increases during puberty, correlating with sexual maturation (45). *ADORA2B* encodes an adenosine receptor and is involved in axon elongation. A previous report showed that *ADORA2B* transcripts were significantly downregulated in GnRH neurons during proestrus (46), while it has not been studied in pituitary. Therefore, these newly defined genes may influence the electrophysiology of puberty in hypothalamus and pituitary gland.

TIPIN is part of the replisome complex and binds the replication fork-protection complex TIMELESS, which is involved in circadian rhythm regulation (47). Circadian rhythms are well known to play key roles in animal reproduction (48). The existing body of research suggests that during the chronotype and the circadian timekeeping system change during the puberty, including endogenous rhythm period and sensitivity to environmental time cues (49). The results of several studies have established that melatonin promotes follicle-stimulating hormone in the pituitary gland and increases serum estrogen levels, thereby accelerating the onset of puberty (50). Those studies indirectly support a potential role of *TIPIN* in AAM.

CPSF recognizes the AAUAAA signal in pre-mRNA and interacts with other factors to facilitate RNA cleavage and the poly(A) addition. CPSF1 is the largest subunit of the CPSF complex. Previous data established that CPSF1 may promote ovarian cancer (51), cell proliferation, and triple-negative breast cancer (52). However, women have a higher risk for developing autoimmune diseases than men, which is attributed to sweeping endocrinological changes during puberty that considerably affect the immune system (53). CPSF1 was previously found to have a high affinity for HLA molecules (54). Thus, *CPSF1* may be linked to the immune response in adolescent girls.

We extended the well-established GSEA approach to detect associations between environmental chemicals and AAM using published GWAS summary data sets and identified 120 chemicals, including drugs, organic compounds, inorganic compounds, plant extracts, nutrients, phenols, plasticizers, pesticides, herbicides, pollutants, and heavy metals. Aromatase is a rate-limiting enzyme in the conversion of androgens to estrogens, and letrozole is a selective aromatase inhibitor (55). Letrozole has been used to treat McCune–Albright syndrome, which has been associated with precocious puberty in girls (56) and delayed growth and puberty in boys (57). Further, fluoxetine is drug that is commonly used to treat adults with depressive disorders (58). The effect of fluoxetine on puberty has been a controversial issue. The results of one study demonstrated that fluoxetine exposure *in utero* delays puberty onset in female rats (59), whereas other data suggested that fluoxetine exposure during gestation did not alter plasma estrogen concentrations in peripubertal offspring (60).

Some plant extracts act as EEDs, as they are among a group of secondary metabolites with chemical structures similar to those of endogenous hormones. Isoflavones are phytoestrogens that are mainly produced in soybeans and can promote advancement of the vaginal opening in female rats after exogenous supplementation (61). Galangin is a naturally occurring flavonoid that inhibits the effects of flavonoids on human cytochrome P450 (62). Galangin was found to inhibit the aryl hydrocarbon receptor and is considered a potential drug for treating breast cancer (63, 64). Notable health benefits have also been reported for galangin (65), and previous data suggest that galangin is useful for treating precocious puberty. Zearalenone (ZEN) is a non-steroidal mycoestrogen that can exert adverse endocrine effects in mammals (66). To date, there is little agreement regarding the effect of ZEN on puberty. One group found that ZEN might trigger central precocious puberty (CPP) development in girls (67), whereas another group found an association between ZEN and normal pubertal development in adolescent girls (68).

A growing body of literature has associated being overweight or obese with early puberty (69); overweight and obese girls undergo menarche earlier than normal-weight girls (70). Several cross-sectional data have suggested that a substantial proportion of girls with CPP have hypertriglyceridemia (71). Cholesterol is an endogenous ligand of estrogen-related receptor alpha (72) that plays a role in activating estrogen receptors (73). Based on these data, we can infer that a high-cholesterol diet may also be a risk factor for precocious puberty. Plasticizers are additives used to produce or promote plasticity and flexibility in plastics, which are commonly used in everyday life. Further, parabens have been added to personal care products as antimicrobial preservatives (74), and methylparaben exposure increases glandular tissue sizes during critical developmental windows (75). A large survey showed that elevated phthalate metabolite levels pose potentially high health risks to children (76). In addition, monobutyl phthalate exposure might be associated with a risk for sexual precocity in girls (77). Animal experiments have demonstrated that two alkylphenols (4-nonylphenol and 4-tert-octylphenol) modify hormone biosynthesis and delay the onset of puberty (78).

This study has some limitations. First, the pooled GWAS data were obtained from the United Kingdom Biobank, and the study subjects were predominantly from European populations; these aspects affect the extrapolation of the results. Therefore, our results should be used cautiously when studying AAM in other populations. Second, although some of the genes we screened have been confirmed in other studies, our enrichment analysis used genes that did not pass correction for multiple testing (P <0.05), which may introduce some false positives so it requires caution when using our results. And some genes related to AAM susceptibility identified here have not been verified *via* molecular biology experiments, which should be performed in future studies. Further, some EEDs identified in this study were previously demonstrated to play a role in AAM, whereas others are not yet validated, which will require more clinical observations and cohort studies. However, to the best of our knowledge, this is the first large study using CGSEA to identify candidate EEDs related to AAM. Our TWAS analysis detected AAM-associated genes at the DNA level, and our CGSEA extended the classic GSEA approach to detect associations between environmental chemicals and AAM.

## Conclusion

In this study, we aimed to determine the influences of genetic and environmental factors on AAM. Therefore, we performed a TWAS and CGSEA related to AAM and identified multiple AAM-associated genes and EEDs. The results of this study expand our understanding of the genetic and environmental factors affecting the timing of female puberty.

## Data Availability Statement

The original contributions presented in the study are included in the article/**Supplementary Material**. Further inquiries can be directed to the corresponding author.

## Author Contributions

Author ML and RF collected and processed the data, as well as wrote this article. YQ and HD provided language help and writing assistance. BL proofreaded the article. CY and YX designed the study. All authors contributed to the article and approved the submitted version.

## Conflict of Interest

The authors declare that the research was conducted in the absence of any commercial or financial relationships that could be construed as a potential conflict of interest.

## Publisher’s Note

All claims expressed in this article are solely those of the authors and do not necessarily represent those of their affiliated organizations, or those of the publisher, the editors and the reviewers. Any product that may be evaluated in this article, or claim that may be made by its manufacturer, is not guaranteed or endorsed by the publisher.

## References

[1] Herting MM Sowell ER. Puberty and Structural Brain Development in Humans. Front Neuroendocrinol (2017) 44:122–37. doi: 10.1016/j.yfrne.2016.12.003 PMC5612369 28007528

[2] Villamor E Jansen EC. Nutritional Determinants of the Timing of Puberty. Annu Rev Public Health (2016) 37:33–46. doi: 10.1146/annurev-publhealth-031914-122606 26789387

[3] Dvornyk V Waqar ul H. Genetics of Age at Menarche: A Systematic Review. Hum Reprod Update (2012) 18(2):198–210. doi: 10.1093/humupd/dmr050 22258758

[4] Avendano MS Vazquez MJ Tena-Sempere M. Disentangling Puberty: Novel Neuroendocrine Pathways and Mechanisms for the Control of Mammalian Puberty. Hum Reprod Update (2017) 23(6):737–63. doi: 10.1093/humupd/dmx025 28961976

[5] Marks KJ Howards PP Smarr MM Flanders WD Northstone K Daniel JH. Prenatal Exposure to Mixtures of Persistent Endocrine Disrupting Chemicals and Early Menarche in a Population-Based Cohort of British Girls. Environ Pollut (2021) 276:116705. doi: 10.1016/j.envpol.2021.116705 33592441 PMC8111784

[6] Lopez-Rodriguez D Franssen D Bakker J Lomniczi A Parent AS. Cellular and Molecular Features of EDC Exposure: Consequences for the GnRH Network. Nat Rev Endocrinol (2021) 17(2):83–96. doi: 10.1038/s41574-020-00436-3 33288917

[7] La Merrill MA Vandenberg LN Smith MT Goodson W Browne P Patisaul HB. Consensus on the Key Characteristics of Endocrine-Disrupting Chemicals as a Basis for Hazard Identification. Nat Rev Endocrinol (2020) 16(1):45–57. doi: 10.1038/s41574-019-0273-8 31719706 PMC6902641

[8] Oskar S Wolff MS Teitelbaum SL Stingone JA. Identifying Environmental Exposure Profiles Associated With Timing of Menarche: A Two-Step Machine Learning Approach to Examine Multiple Environmental Exposures. Environ Res (2021) 195:110524. doi: 10.1016/j.envres.2020.110524 33249040 PMC8673778

[9] Lopez-Rodriguez D Aylwin CF Delli V Sevrin E Campanile M Martin M. Multi- and Transgenerational Outcomes of an Exposure to a Mixture of Endocrine-Disrupting Chemicals (EDCs) on Puberty and Maternal Behavior in the Female Rat. Environ Health Perspect (2021) 129(8):87003. doi: 10.1289/EHP8795 34383603 PMC8360047

[10] Wu C Tan S Liu L Cheng S Li P Li W. Transcriptome-Wide Association Study Identifies Susceptibility Genes for Rheumatoid Arthritis. Arthritis Res Ther (2021) 23(1):38. doi: 10.1186/s13075-021-02419-9 33482886 PMC7821659

[11] Lin WD Cheng CF Wang CH Liang WM Chen CH Hsieh AR. Genetic Factors of Idiopathic Central Precocious Puberty and Their Polygenic Risk in Early Puberty. Eur J Endocrinol (2021) 185(4):441–51. doi: 10.1530/EJE-21-0424 34288885

[12] Albert FW Kruglyak L. The Role of Regulatory Variation in Complex Traits and Disease. Nat Rev Genet (2015) 16(4):197–212. doi: 10.1038/nrg3891 25707927

[13] Gusev A Ko A Shi H Bhatia G Chung W Penninx BW. Integrative Approaches for Large-Scale Transcriptome-Wide Association Studies. Nat Genet (2016) 48(3):245–52. doi: 10.1038/ng.3506 PMC4767558 26854917

[14] Shi J Wu L Li B Lu Y Guo X Cai Q. Transcriptome-Wide Association Study Identifies Susceptibility Loci and Genes for Age at Natural Menopause. Reprod Sci (2019) 26(4):496–502. doi: 10.1177/1933719118776788 29848177 PMC6421625

[15] Abreu AP Kaiser UB. Pubertal Development and Regulation. Lancet Diabetes Endocrinol (2016) 4(3):254–64. doi: 10.1016/S2213-8587(15)00418-0 PMC5192018 26852256

[16] Day FR Thompson DJ Helgason H Chasman DI Finucane H Sulem P. Genomic Analyses Identify Hundreds of Variants Associated With Age at Menarche and Support a Role for Puberty Timing in Cancer Risk. Nat Genet (2017) 49(6):834–41. 10.1038/ng.3841 PMC5841952 28436984

[17] Zhang Y Quick C Yu K Barbeira A Consortium GT Luca F. PTWAS: Investigating Tissue-Relevant Causal Molecular Mechanisms of Complex Traits Using Probabilistic TWAS Analysis. Genome Biol (2020) 21(1):232. doi: 10.1186/s13059-020-02026-y 32912253 PMC7488550

[18] Zhou X Carbonetto P Stephens M. Polygenic Modeling With Bayesian Sparse Linear Mixed Models. PLoS Genet (2013) 9(2):e1003264. doi: 10.1371/journal.pgen.1003264 23408905 PMC3567190

[19] Kanehisa M Goto S. KEGG: Kyoto Encyclopedia of Genes and Genomes. Nucleic Acids Res (2000) 28(1):27–30. doi: 10.1093/nar/28.1.27 10592173 PMC102409

[20] Hill DP Blake JA Richardson JE Ringwald M. Extension and Integration of the Gene Ontology (GO): Combining GO Vocabularies With External Vocabularies. Genome Res (2002) 12(12):1982–91. doi: 10.1101/gr.580102 PMC187579 12466303

[21] Jensen LJ Kuhn M Stark M Chaffron S Creevey C Muller J. STRING 8–a Global View on Proteins and Their Functional Interactions in 630 Organisms. Nucleic Acids Res (2009) 37(Database issue):D412–6. doi: 10.1093/nar/gkn760 PMC2686466 18940858

[22] Shannon P Markiel A Ozier O Baliga NS Wang JT Ramage D. Cytoscape: A Software Environment for Integrated Models of Biomolecular Interaction Networks. Genome Res (2003) 13(11):2498–504. doi: 10.1101/gr.1239303 PMC403769 14597658

[23] Bader GD Hogue CW. An Automated Method for Finding Molecular Complexes in Large Protein Interaction Networks. BMC Bioinf (2003) 4:2. doi: 10.1186/1471-2105-4-2 PMC149346 12525261

[24] Mattingly CJ Colby GT Rosenstein MC Forrest JN Jr Boyer JL. Promoting Comparative Molecular Studies in Environmental Health Research: An Overview of the Comparative Toxicogenomics Database (CTD). Pharmacogenomics J (2004) 4(1):5–8. doi: 10.1038/sj.tpj.6500225 14735110

[25] Cheng S Ma M Zhang L Liu L Cheng B Qi X. CGSEA: A Flexible Tool for Evaluating the Associations of Chemicals With Complex Diseases. G3 (Bethesda) (2020) 10(3):945–9. doi: 10.1534/g3.119.400945 PMC7056963 31937547

[26] Wang K Li M Bucan M. Pathway-Based Approaches for Analysis of Genomewide Association Studies. Am J Hum Genet (2007) 81(6):1278–83. doi: 10.1086/522374 PMC2276352 17966091

[27] Mooney MA Wilmot B. Gene Set Analysis: A Step-by-Step Guide. Am J Med Genet B Neuropsychiatr Genet (2015) 168(7):517–27. doi: 10.1002/ajmg.b.32328 PMC4638147 26059482

[28] Ziyatdinov A Vázquez-Santiago M Brunel H Martinez-Perez A Aschard H Soria JM. Lme4qtl: Linear Mixed Models With Flexible Covariance Structure for Genetic Studies of Related Individuals. BMC Bioinf (2018) 19(1):68. doi: 10.1186/s12859-018-2057-x PMC5830078 29486711

[29] Stamou MI Balasubramanian R. Hypothalamic Ceramides and the Ovarian Sympathetic System: At the Crossroads of Obesity and Puberty. Cell Metab (2021) 33(1):6–8. doi: 10.1016/j.cmet.2020.11.012 33264644 PMC8939237

[30] Gracia-Tabuenca Z Moreno MB Barrios FA Alcauter S. Development of the Brain Functional Connectome Follows Puberty-Dependent Nonlinear Trajectories. Neuroimage (2021) 229:117769. doi: 10.1016/j.neuroimage.2021.117769 33482398

[31] Navarro VM. Metabolic Regulation of Kisspeptin - the Link Between Energy Balance and Reproduction. Nat Rev Endocrinol (2020) 16(8):407–20. doi: 10.1038/s41574-020-0363-7 PMC8852368 32427949

[32] Perry JR Day F Elks CE Sulem P Thompson DJ Ferreira T. Parent-Of-Origin-Specific Allelic Associations Among 106 Genomic Loci for Age at Menarche. Nature (2014) 514(7520):92–7. 10.1038/nature13545 PMC4185210 25231870

[33] Cousminer DL Berry DJ Timpson NJ Ang W Thiering E Byrne EM. Genome-Wide Association and Longitudinal Analyses Reveal Genetic Loci Linking Pubertal Height Growth, Pubertal Timing and Childhood Adiposity. Hum Mol Genet (2013) 22(13):2735–47. doi: 10.1093/hmg/ddt104 PMC3674797 23449627

[34] Elks CE Perry JRB Sulem P Chasman DI Franceschini N He C. Thirty New Loci for Age at Menarche Identified by a Meta-Analysis of Genome-Wide Association Studies. Nat Genet (2010) 42(12):1077–85. 10.1038/ng.714 PMC3140055 21102462

[35] Mancuso N Shi H Goddard P Kichaev G Gusev A Pasaniuc B. Integrating Gene Expression With Summary Association Statistics to Identify Genes Associated With 30 Complex Traits. Am J Hum Genet (2017) 100(3):473–87. doi: 10.1016/j.ajhg.2017.01.031 PMC5339290 28238358

[36] Cheng TS Day FR Perry JRB Luan J Langenberg C Forouhi NG. Prepubertal Dietary and Plasma Phospholipid Fatty Acids Related to Puberty Timing: Longitudinal Cohort and Mendelian Randomization Analyses. Nutrients (2021) 13(6). doi: 10.3390/nu13061868 PMC8228200 34070864

[37] Kemiläinen H Adam M Mäki-Jouppila J Damdimopoulou P Damdimopoulos AE Kere J. The Hydroxysteroid (17β) Dehydrogenase Family Gene HSD17B12 Is Involved in the Prostaglandin Synthesis Pathway, the Ovarian Function, and Regulation of Fertility. Endocrinology (2016) 157(10):3719–30. doi: 10.1210/en.2016-1252 27490311

[38] Heikelä H Ruohonen ST Adam M Viitanen R Liljenbäck H Eskola O. Hydroxysteroid (17β) Dehydrogenase 12 is Essential for Metabolic Homeostasis in Adult Mice. Am J Physiol Endocrinol Metab (2020) 319(3):E494–e508. doi: 10.1152/ajpendo.00042.2020 32691632

[39] Heras V Castellano JM Fernandois D Velasco I Rodríguez-Vazquez E Roa J. Central Ceramide Signaling Mediates Obesity-Induced Precocious Puberty. Cell Metab (2020) 32(6):951–966.e8. doi: 10.1016/j.cmet.2020.10.001 33080217

[40] Hevir-Kene N Rižner TL. The Endometrial Cancer Cell Lines Ishikawa and HEC-1A, and the Control Cell Line HIEEC, Differ in Expression of Estrogen Biosynthetic and Metabolic Genes, and in Androstenedione and Estrone-Sulfate Metabolism. Chem Biol Interact (2015) 234:309–19. doi: 10.1016/j.cbi.2014.11.015 25437045

[41] Szajnik M Szczepanski MJ Elishaev E Visus C Lenzner D Zabel M. 17β Hydroxysteroid Dehydrogenase Type 12 (HSD17B12) is a Marker of Poor Prognosis in Ovarian Carcinoma. Gynecol Oncol (2012) 127(3):587–94. doi: 10.1016/j.ygyno.2012.08.010 PMC3607433 22903146

[42] Haynes BP Straume AH Geisler J A'Hern R Helle H Smith IE. Intratumoral Estrogen Disposition in Breast Cancer. Clin Cancer Res (2010) 16(6):1790–801. doi: 10.1158/1078-0432.CCR-09-2481 20215536

[43] Conduit SE Davies EM Fulcher AJ Oorschot V Mitchell CA. Superresolution Microscopy Reveals Distinct Phosphoinositide Subdomains Within the Cilia Transition Zone. Front Cell Dev Biol (2021) 9:634649. doi: 10.3389/fcell.2021.634649 33996795 PMC8120242

[44] Guo J Otis JM Suciu SK Catalano C Xing L Constable S. Primary Cilia Signaling Promotes Axonal Tract Development and Is Disrupted in Joubert Syndrome-Related Disorders Models. Dev Cell (2019) 51(6):759–74.e5. doi: 10.1016/j.devcel.2019.11.005 31846650 PMC6953258

[45] Koemeter-Cox AI Sherwood TW Green JA Steiner RA Berbari NF Yoder BK. Primary Cilia Enhance Kisspeptin Receptor Signaling on Gonadotropin-Releasing Hormone Neurons. Proc Natl Acad Sci U S A (2014) 111(28):10335–40. doi: 10.1073/pnas.1403286111 PMC4104922 24982149

[46] Rodolosse A Solymosi N Liposits Z. Altered Expression of Genes Encoding Neurotransmitter Receptors in GnRH Neurons of Proestrous Mice. Front Cell Neurosci (2016) 10:230. doi: 10.3389/fncel.2016.00230 27774052 PMC5054603

[47] Kondratov RV Antoch MP. Circadian Proteins in the Regulation of Cell Cycle and Genotoxic Stress Responses. Trends Cell Biol (2007) 17(7):311–7. doi: 10.1016/j.tcb.2007.07.001 17644383

[48] Basili D Gioacchini G Todisco V Candelma M Marisaldi L Pappalardo L. Opsins and Gonadal Circadian Rhythm in the Swordfish (Xiphias Gladius) Ovary: Their Potential Roles in Puberty and Reproductive Seasonality. Gen Comp Endocrinol (2021) 303:113707. doi: 10.1016/j.ygcen.2020.113707 33387470

[49] Hagenauer MH Lee TM. The Neuroendocrine Control of the Circadian System: Adolescent Chronotype. Front Neuroendocrinol (2012) 33(3):211–29. doi: 10.1016/j.yfrne.2012.04.003 PMC4762453 22634481

[50] Yang C Ran Z Liu G Hou R He C Liu Q. Melatonin Administration Accelerates Puberty Onset in Mice by Promoting FSH Synthesis. Molecules (2021) 26(5). doi: 10.3390/molecules26051474 PMC7963190 33803091

[51] Zhang B Liu Y Liu D Yang L. Targeting Cleavage and Polyadenylation Specific Factor 1 via shRNA Inhibits Cell Proliferation in Human Ovarian Cancer. J Biosci (2017) 42(3):417–25. doi: 10.1007/s12038-017-9701-x 29358555

[52] Wang L Lang GT Xue MZ Yang L Chen L Yao L. Dissecting the Heterogeneity of the Alternative Polyadenylation Profiles in Triple-Negative Breast Cancers. Theranostics (2020) 10(23):10531–47. doi: 10.7150/thno.40944 PMC7482814 32929364

[53] Desai MK Brinton RD. Autoimmune Disease in Women: Endocrine Transition and Risk Across the Lifespan. Front Endocrinol (Lausanne) (2019) 10:265. doi: 10.3389/fendo.2019.00265 31110493 PMC6501433

[54] Noblejas-López MDM Nieto-Jiménez C Morcillo García S Pérez-Peña J Nuncia-Cantarero M Andrés-Pretel F. Expression of MHC Class I, HLA-A and HLA-B Identifies Immune-Activated Breast Tumors With Favorable Outcome. Oncoimmunology (2019) 8(10):e1629780. doi: 10.1080/2162402X.2019.1629780 31646075 PMC6791424

[55] Wit JM Hero M Nunez SB. Aromatase Inhibitors in Pediatrics. Nat Rev Endocrinol (2011) 8(3):135–47. doi: 10.1038/nrendo.2011.161 22024975

[56] Estrada A Boyce AM Brillante BA Guthrie LC Gafni RI Collins MT. Long-Term Outcomes of Letrozole Treatment for Precocious Puberty in Girls With McCune-Albright Syndrome. Eur J Endocrinol (2016) 175(5):477–83. doi: 10.1530/EJE-16-0526 PMC5066167 27562402

[57] Kohva E Varimo T Huopio H Tenhola S Voutilainen R Toppari J. Anti-Müllerian Hormone and Letrozole Levels in Boys With Constitutional Delay of Growth and Puberty Treated With Letrozole or Testosterone. Hum Reprod (2020) 35(2):257–64. doi: 10.1093/humrep/dez231 PMC7048712 31958337

[58] Cipriani A Furukawa TA Salanti G Chaimani A Atkinson LZ Ogawa Y. Comparative Efficacy and Acceptability of 21 Antidepressant Drugs for the Acute Treatment of Adults With Major Depressive Disorder: A Systematic Review and Network Meta-Analysis. Lancet (2018) 391(10128):1357–66. doi: 10.1016/S0140-6736(17)32802-7 PMC5889788 29477251

[59] Dos Santos AH Vieira ML de Azevedo Camin N Anselmo-Franci JA Ceravolo GS Pelosi GG. In Utero and Lactational Exposure to Fluoxetine Delays Puberty Onset in Female Rats Offspring. Reprod Toxicol (2016) 62:1–8. doi: 10.1016/j.reprotox.2016.04.006 27094375

[60] Barbosa MA Veríssimo LF Gerardin DCC Pelosi GG Ceravolo GS Moreira EG. Maternal Exposure to Fluoxetine During Gestation and Lactation Does Not Alter Plasma Concentrations of Testosterone, Oestrogen or Corticosterone in Peripubertal Offspring. Reprod Fertil Dev (2019) 31(5):1002–8. doi: 10.1071/RD18279 30786956

[61] Sleiman HK de Oliveira JM Langoni de Freitas GB. Isoflavones Alter Male and Female Fertility in Different Development Windows. BioMed Pharmacother (2021) 140:111448. doi: 10.1016/j.biopha.2021.111448 34130202

[62] Kao YC Zhou C Sherman M Laughton CA Chen S. Molecular Basis of the Inhibition of Human Aromatase (Estrogen Synthetase) by Flavone and Isoflavone Phytoestrogens: A Site-Directed Mutagenesis Study. Environ Health Perspect (1998) 106(2):85–92. doi: 10.1289/ehp.9810685 PMC1533021 9435150

[63] Murray TJ Yang X Sherr DH. Growth of a Human Mammary Tumor Cell Line is Blocked by Galangin, a Naturally Occurring Bioflavonoid, and is Accompanied by Down-Regulation of Cyclins D3, E and A. Breast Cancer Res (2006) 8(2):R17. doi: 10.1186/bcr1391 16569260 PMC1557718

[64] Donovan MG Selmin OI Doetschman TC Romagnolo DF. Epigenetic Activation of BRCA1 by Genistein In Vivo and Triple Negative Breast Cancer Cells Linked to Antagonism Toward Aryl Hydrocarbon Receptor. Nutrients (2019) 11(11). doi: 10.3390/nu11112559 PMC6893467 31652854

[65] Xuan H Ou A Hao S Shi J Jin X. Galangin Protects Against Symptoms of Dextran Sodium Sulfate-Induced Acute Colitis by Activating Autophagy and Modulating the Gut Microbiota. Nutrients (2020) 12(2). doi: 10.3390/nu12020347 PMC7071155 32013062

[66] Ali N Degen GH. Urinary Biomarkers of Exposure to the Mycoestrogen Zearalenone and its Modified Forms in German Adults. Arch Toxicol (2018) 92(8):2691–700. doi: 10.1007/s00204-018-2261-5 29980802

[67] Massart F Meucci V Saggese G Soldani G. High Growth Rate of Girls With Precocious Puberty Exposed to Estrogenic Mycotoxins. J Pediatr (2008) 152(5):690–5, 695.e1. doi: 10.1016/j.jpeds.2007.10.020 18410776

[68] Rivera-Núñez Z Barrett ES Szamreta EA Shapses SA Qin B Lin Y. Urinary Mycoestrogens and Age and Height at Menarche in New Jersey Girls. Environ Health (2019) 18(1):24. doi: 10.1186/s12940-019-0464-8 30902092 PMC6431018

[69] Brix N Ernst A Lauridsen LLB Parner ET Arah OA Olsen J. Childhood Overweight and Obesity and Timing of Puberty in Boys and Girls: Cohort and Sibling-Matched Analyses. Int J Epidemiol (2020) 49(3):834–44. doi: 10.1093/ije/dyaa056 PMC7394964 32372073

[70] Ortega MT McGrath JA Carlson L Flores Poccia V Larson G Douglas C. Longitudinal Investigation of Pubertal Milestones and Hormones as a Function of Body Fat in Girls. J Clin Endocrinol Metab (2021) 106(6):1668–83. doi: 10.1210/clinem/dgab092 PMC8118584 33630047

[71] Zurita-Cruz JN Villasís-Keever MA Manuel-Apolinar L Damasio-Santana L Gutierrez-Gonzalez A Wakida-Kusunoki G. Altered Cardiometabolic Profile in Girls With Central Precocious Puberty and Adipokines: A Propensity Score Matching Analysis. Cytokine (2021) 148:155660. doi: 10.1016/j.cyto.2021.155660 34334260

[72] Wei W Schwaid AG Wang X Wang X Chen S Chu Q. Ligand Activation of Errα by Cholesterol Mediates Statin and Bisphosphonate Effects. Cell Metab (2016) 23(3):479–91. doi: 10.1016/j.cmet.2015.12.010 PMC4785078 26777690

[73] Li D Cai Y Teng D Li W Tang Y Liu G. Computational Insights Into the Interaction Mechanisms of Estrogen-Related Receptor Alpha With Endogenous Ligand Cholesterol. Chem Biol Drug Des (2019) 94(1):1316–29. doi: 10.1111/cbdd.13506 30811808

[74] Jensen TK Andersson AM Main KM Johannsen TH Andersen MS Kyhl HB. Prenatal Paraben Exposure and Anogenital Distance and Reproductive Hormones During Mini-Puberty: A Study From the Odense Child Cohort. Sci Total Environ (2021) 769:145119. doi: 10.1016/j.scitotenv.2021.145119 33477047

[75] Gopalakrishnan K Teitelbaum SL Lambertini L Wetmur J Manservisi F Falcioni L. Changes in Mammary Histology and Transcriptome Profiles by Low-Dose Exposure to Environmental Phenols at Critical Windows of Development. Environ Res (2017) 152:233–43. doi: 10.1016/j.envres.2016.10.021 PMC5135583 27810681

[76] Yao Y Chen D Wu Y Zhou L Cheng J Li Y. Urinary Phthalate Metabolites in Primary School Starters in Pearl River Delta, China: Occurrences, Risks and Possible Sources. Environ Res (2019) 179(Pt B):108853. doi: 10.1016/j.envres.2019.108853 31678724

[77] Wen Y Liu SD Lei X Ling YS Luo Y Liu Q. Association of PAEs With Precocious Puberty in Children: A Systematic Review and Meta-Analysis. Int J Environ Res Public Health (2015) 12(12):15254–68. doi: 10.3390/ijerph121214974 PMC4690910 26633449

[78] Patiño-García D Cruz-Fernandes L Buñay J Palomino J Moreno RD. Reproductive Alterations in Chronically Exposed Female Mice to Environmentally Relevant Doses of a Mixture of Phthalates and Alkylphenols. Endocrinology (2018) 159(2):1050–61. doi: 10.1210/en.2017-00614 29300862

